# Anatomy and variations of the carina and its cartilaginous makeup: a cadaveric study

**DOI:** 10.1007/s00276-025-03579-y

**Published:** 2025-02-04

**Authors:** Chung Yoh Kim, Kristen Rizzuto, Daniel Tahan, Devendra Shekhawat, Rarinthorn Samrid, Yoko Tabira, Stephen J. Bordes, Joe Iwanaga, R. Shane Tubbs

**Affiliations:** 1 Department of Neurosurgery, Tulane Center for Clinical Neurosciences, Tulane University School of Medicine, 131 S. Robertson St., Suite 1300, New Orleans, LA 70112 USA; 2 Department of Anatomy, Dongguk University School of Medicine, Gyeongju, Republic of Korea; 3 Tulane University School of Medicine, New Orleans, LA USA; 4 Department of Anatomy, Faculty of Medicine, Khon Kaen University, Khon Kaen, Thailand; 5 Division of Gross and Clinical Anatomy, Department of Anatomy, Kurume University School of Medicine, Kurume, Fukuoka Japan; 6 Department of Surgery, Louisiana State University Health Sciences Center, New Orleans, LA USA; 7 Department of Neurology, Tulane Center for Clinical Neurosciences, Tulane University School of Medicine, New Orleans, LA USA; 8 Department of Structural & Cellular Biology, Tulane University School of Medicine, New Orleans, LA USA; 9 Department of Neurosurgery and Ochsner Neuroscience Institute, Ochsner Health System, New Orleans, LA USA; 10 Department of Anatomical Sciences, St. George’s University, St. George’s, Grenada; 11 Department of Surgery, Tulane University School of Medicine, New Orleans, LA USA; 12 University of Queensland, Brisbane, Australia; 13 Dental and Oral Medical Center, Kurume University School of Medicine, 67 Asahi-machi, Kurume, Fukuoka Japan

**Keywords:** Cartilage, Trachea, Bronchus, Carinal cartilage, Respiratory, Aspiration

## Abstract

**Purpose:**

The carina, located at the bifurcation of the trachea, has been regarded as a part of the trachea. Although clinically useful as an anatomical landmark, studies of its detailed morphology are lacking in the literature.

**Methods:**

The distal trachea and left and right main bronchi were harvested from 32 cadavers and the carina studied using microsurgical dissection, endoscopy, micro-CT, and histology.

**Results:**

The right bronchial cartilages were most commonly involved in forming the carina (72.41%), and the left bronchial cartilages were the second most commonly involved (37.93%). The carinal cartilages were slightly deviated to the left of midline in 4.37%. Micro-CT clearly identified the contributions to the carinal cartilages.

**Conclusion:**

Although the carina has been regarded as a part of the distal trachea, the present study found that most of the carinal cartilages were composed of the most inferior tracheal ring or bronchial cartilage(s). The right main bronchial cartilage was the most common contributor, and the left main bronchus was the second most common contributor. Additional knowledge of this structure can benefit patient care.

## Introduction

The carina is generally regarded as a cartilaginous projection from the most inferior tracheal ring, protruding internally at the tracheobronchial junction and seen as a keel-like structure during bronchoscopy. In the second edition of *Terminologia Anatomica* [3], the carina is listed as “carina tracheae”, implying that it is related only to the trachea.

However, the carina is clinically important in various diagnoses, surgeries, and interventions. Morphological changes in the carina, such as increased subcarinal angles, are important diagnostic signs for bronchogenic carcinoma, as metastasized subcarinal lymph nodes can be enlarged [13]. Although tumors in the distal trachea and carina are less common, carina resection and reconstruction are occasionally necessary, presenting challenges due to technical difficulties and poor cartilage healing [11]. A Y-shaped stent near the carina may be used to address tracheobronchial stenosis [10]. Furthermore, since the mucous membrane covering the carina is one of the most sensitive areas of the tracheobronchial tree associated with the cough reflex, the carina is used as a landmark for endotracheal tube placement and as an indicator of the midline e. g., midline shift [2].

Despite the significant role of the carina in pulmonology and thoracic imaging and surgery, its comprehensive anatomy and variations have yet to be extensively studied. Therefore, this cadaveric study examined the comprehensive anatomy and variations of the carina and its contributing cartilage.

## Materials and methods

### Microsurgical dissection

**Fig. 1:**
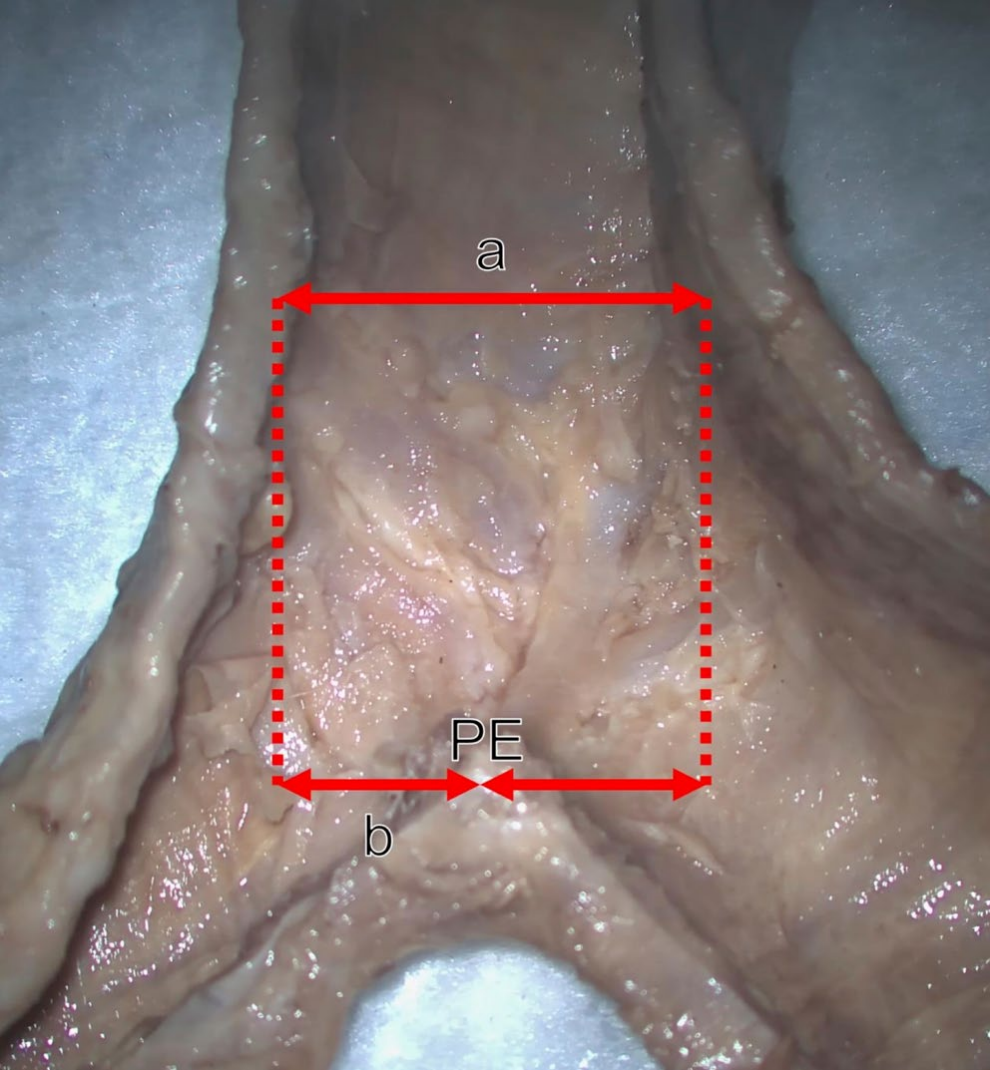
Measurement of carinal cartilage deviation (posterior view). To determine deviation of the carina, the distal tracheal diameter (**a**), and distance between the posterior endpoint of the carina (PE) and left lateral border of the distal trachea (**b**) were measured with microcalipers

The trachea and left and right main stem bronchi were harvested from 32 cadavers (15 males and 17 females). Twenty-nine specimens were dissected, using a surgical microscope (Zeiss, Oberkochen, Germany) to examine the cartilaginous contribution to carinal cartilage. The posterior wall of the trachea and bronchi was incised following the lateral border of the bilateral main bronchi to the trachea. The mucosal surface of the inner surface of the airway was carefully dissected away to visualize the carinal and other cartilages of the trachea and main bronchi. The tracheal and bilateral main bronchial cartilages were examined to determine their contribution to the carinal cartilage formation. The length and width of the carina were measured using digital micro calipers (Mitutoyo, Kanagawa, Japan). To determine the deviation of the carina in the airway, the diameter of the distal trachea and the distance between the posterior end of the carina and the lateral border of the distal trachea were measured (Fig. 1). The present study was performed in accordance with the requirements of the Declaration of Helsinki (64th WMA General Assembly, Fortaleza, Brazil, October 2013). The authors affirm their commitment to adhering rigorously to both local and international ethical protocols and regulations governing the utilization of human cadaveric donors for anatomical research [7].

### Micro-CT and endoscopic image acquisition

Three specimens were randomly chosen and examined with micro-CT. Images were taken using a micro-CT system (Quantum GX2 Micro CT, PerkinElmer, Waltham, Massachusetts, USA). The exposure volume was set at 36 mm diameter and 36 mm height. CT scans were obtained with a voxel size of 0.144 mm. The scan was set at 90 kV and 88 µA. The axial images were transmitted in the digital imaging and communication in medicine (DICOM) format, and 2D images of the carina were then reconstructed using the DICOM viewer (OsiriX, Rosset, et al., 2004). For endoscopic images (Teslong, Irvine, California, USA)), the internal surface of the distal trachea and position of the carina were photographed.

### Histological analysis

The distal tracheas, including the carina and the left and right main bronchi, were cut in axial, coronal, and sagittal planes and embedded in paraffin. A microtome was used to cut 5 μm sections, which were then stained with hematoxylin and eosin, and Masson’s trichrome solutions [1]. The tissues were histologically observed under a light microscope (EVOS FL auto-imaging system, ThermoFisher Scientific, Waltham, Massachusetts, USA), the collagen fibers appeared blue in Masson’s trichrome staining.

## Results

### Contributions to carinal cartilage formation

**Fig. 2:**
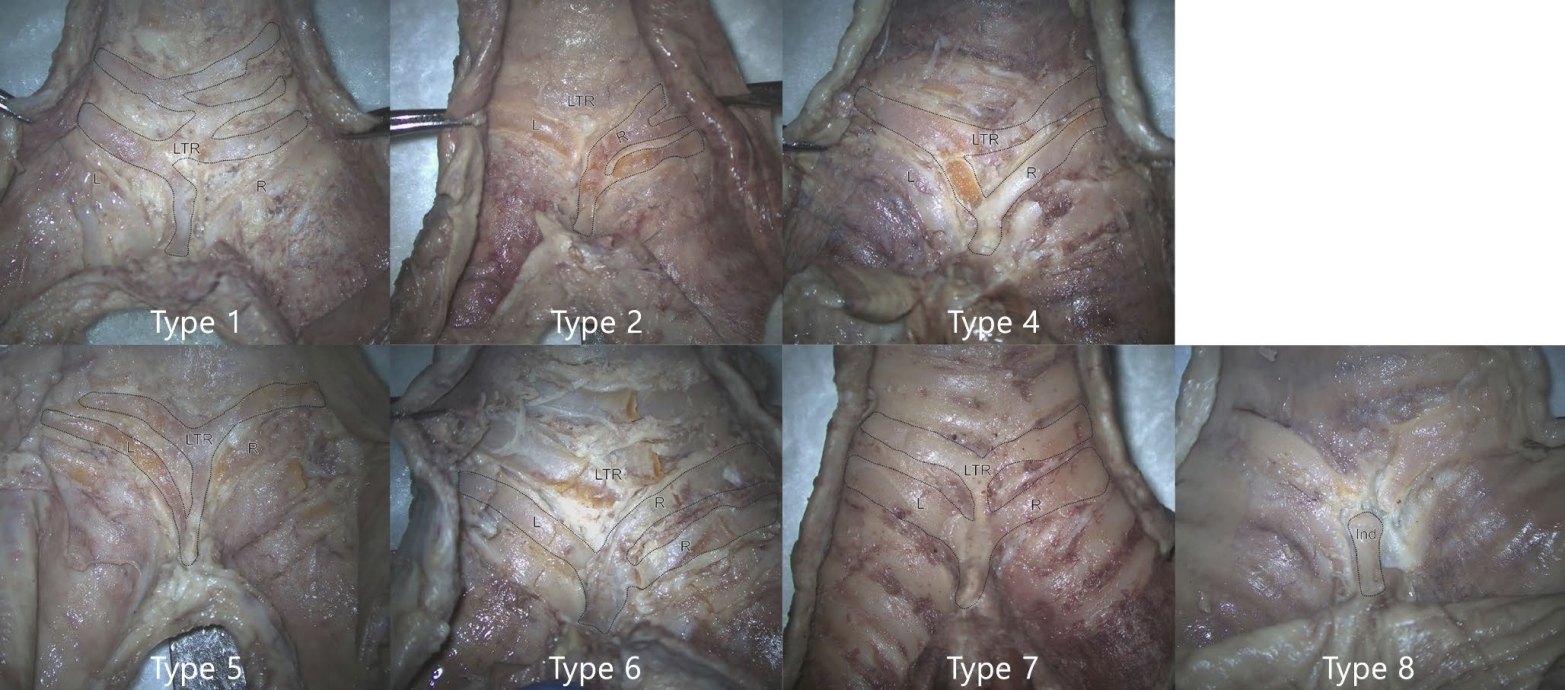
Different types of carinal cartilage based on cartilaginous contributions (posterior view). Note that a type 3 carinal cartilage was not observed in this study. Depending on the cartilaginous contributions to the carina, seven types of carina were classified: Ind, independent carina; L, left main bronchial cartilage; LTR, last tracheal ring; R, right main bronchial cartilage

The inferomost tracheal ring, right and left main bronchial rings, contributed to the formation of the carinal cartilages. The components contributed solely to the formation of carinal cartilage or with the other components. Based on the combinations of cartilaginous contributions for carinal cartilage, eight types of carina were classified (Fig. 2; Table 1). The most common type was carinal cartilage formed by one or more right main bronchial cartilages (type 2, 34.48%). The second common type was made by bilateral main bronchial cartilages (type 6, 24.14%).

**Table 1:** Classification of carina based on cartilaginous contributions

Type	Cartilage contributions	Number of specimens	%
1	Last tracheal ring(s)	3	10.34
2	Right bronchial ring(s)	10	34.48
3	Left bronchial ring(s)	0	0.00
4	Last tracheal ring and right bronchial ring(s)	2	6.90
5	Last tracheal ring and left bronchial ring(s)	2	6.90
6	Bilateral bronchial rings	7	24.14
7	Last tracheal ring and bilateral bronchial rings	2	6.90
8	Independent formation	3	10.34
Total		29	100.00

Consequently, carinal cartilages formed only by tracheal rings, and the ones formed independently without any contribution of components were thirdly common (type 1, 10.34%). Interestingly, this study did not observe carinal cartilage composed solely of left bronchial cartilage (type 3). The left bronchial cartilages were observed to contribute to carinal cartilage with other components. The components are sometimes communicated with different kinds of components. For example, among the type 6, the most inferior tracheal rings were sometimes connected to bilateral bronchial rings but not to carinal cartilage. In the case of type 8, independent carinal cartilage, although components run near the carinal cartilage, fibrous tissues between the carina and components could be identified outside the clean boundaries of the carinal cartilage.

### Measurement of carinal cartilage and deviation of the carina

The carina’s average linear length and thickness were 14.42 (SD: 3.32) mm and 1.89 (SD: 0.35) mm, respectively. The average distal tracheal diameter was 18.25 (SD: 4.28) mm. On average, the distance between the carina’s posterior end and the distal trachea’s left lateral border was 8.33 (SD: 2.17) mm. Among 29 specimens, 25 carinae were left deviated about 4.37% from the midpoint of the distal trachea diameter, which means the lumen of the right main bronchus was larger than the left.

### Micro-CT

**Fig. 3:**
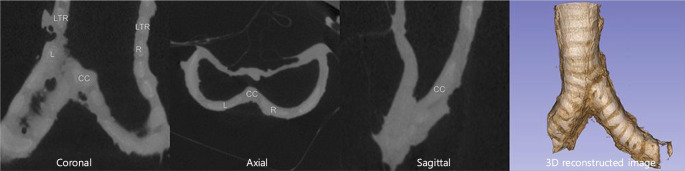
Micro-computed tomography of the carina. The cartilaginous contributions to carina could be identified in the micro-CT; CC, carinal cartilages; L left main bronchial cartilage; LTR, last tracheal ring; R, right main bronchial cartilage

On micro-CT, the tracheal and bronchial cartilages and surrounding soft tissues were visualized clearly and in detail and were gray in color (Fig. 3). In the distal part of the main bronchi, calcification of cartilages were observed as bright white. The carinal cartilages and their cartilaginous contributions were seen at the junction of the distal trachea and left and right main bronchi. In the three-dimensional reconstructed models of the carina, the cartilaginous shape could be recognized even if when covered with soft tissues (Fig. 3).

### Endoscopy

**Fig. 4:**
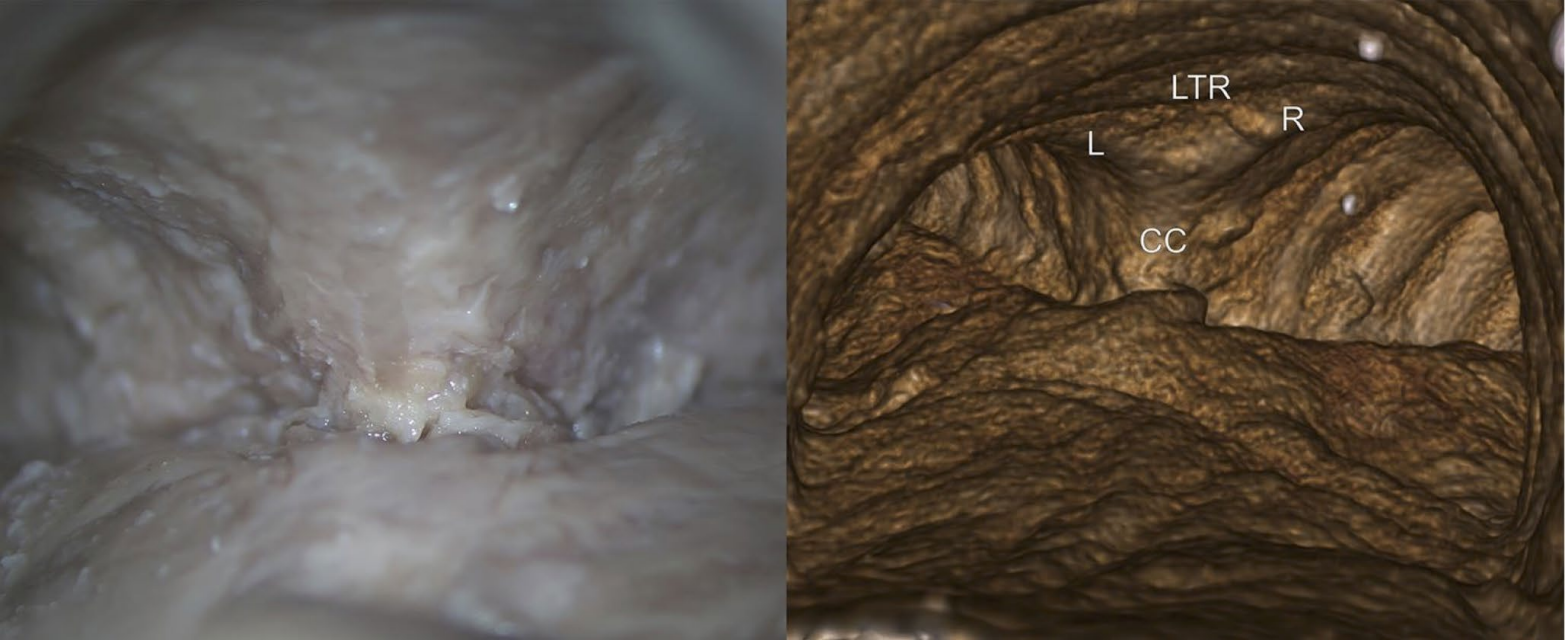
Endoscopic images and three-dimensionally reconstructed images of the carina On micro-CT, the contributing cartilage(s) to the carina were clear; CC, carinal cartilages; L, left main bronchial cartilage; LTR, last tracheal ring; R, right main bronchial cartilage

The endoscopic images showed the internal view of the carina but not the details of the carinal cartilage or its makeup (Fig. 4). These images were limited to a superior view of the carina.

### Histology

**Fig. 5:**
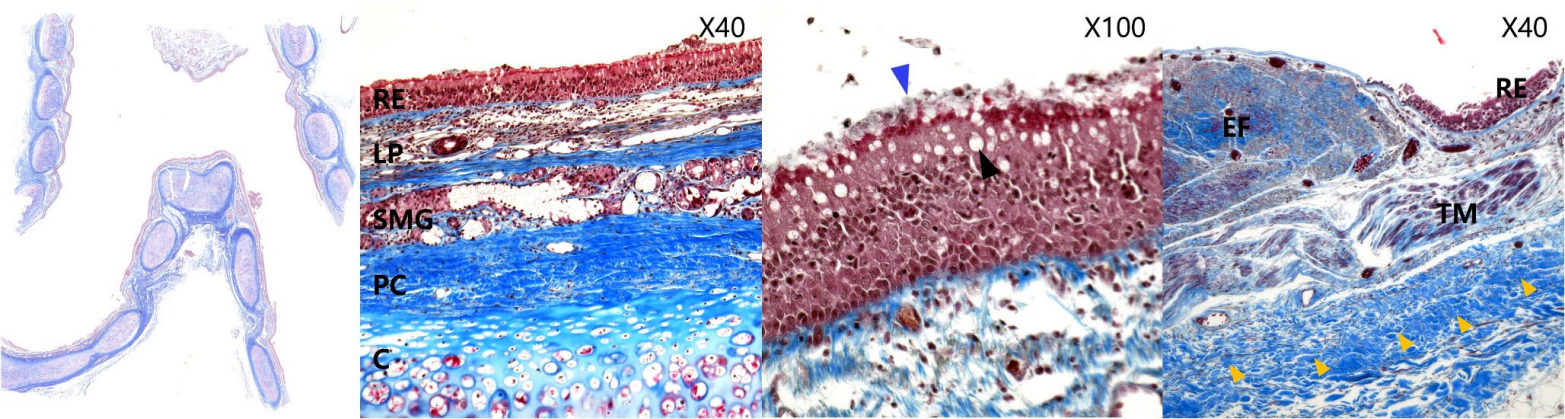
Histological images of the carina. The carina showed the same histology as the adjacent respiratory tract with Masson’s trichrome (40x and100x). The epithelial layer showed respiratory epithelium (RE) with cilia (blue arrowhead) and goblet cells (black arrowhead). In the membranous part of the carina, extensive bundles of elastic fibers (EF) were observed below the epithelial layer; C, chondrium; EF, elastic fibers; LP, lamina propria; PC, perichondrium; RE, respiratory epithelium; SMC, seromucous gland; TM, trachealis muscle and the bundles of collagen fibers (yellow arrowhead)

The distal tracheas, including the carinal cartilage, were histologically examined in three different planes, i. e., axial, coronal, and sagittal planes. These sections showed the same histological characteristics as the distal trachea. Pseudostratified columnar epithelium (respiratory epithelium), with cilia on its apical surface, covered most of the internal surface of the trachea and carina (Fig. 5). Goblet cells and the basement membrane were visible in the epithelial layer. Seromucous glands were observed in the submucosa layer, and the perichondrium showed thick, dense connective tissues below the glandular layer. Chondrocytes with lacuna were visible and indicative of hyaline cartilage, but no elastic fibers were identified.

On the membranous part of the distal trachea, respiratory epithelium also covered its surface (Fig. 5). However, below the basement membrane, extensive bundles of collagen fibers were observed in the submucosa [9]. These collagen fiber bundles could be observed at the end of the carinal cartilages. The trachealis muscles ran transversely, and dense connective tissues were observed under the bundles of collagen fibers.

## Discussion

The term “carina,” i. e., carina and carinal cartilage, has been used to describe different anatomical areas/structures in the literature. This has caused discrepancies and confusion in the results of previous studies. Therefore, in the present study, we used the term “carina” for the description of the distal trachea between the openings of the main bronchi and the term “carinal cartilage” as specifically for the cartilage that forms the carina.

Examination of the composition of the cartilages contributing to the carina have scarcely been studied since Vanpeperstraete’s publication [15]. Vanpeperstraete summarized cartilaginous continuity between the trachea and bronchi. Interestingly, our results differed from his study [15]. From previous study, the majority of the specimens (67/100) were defined as “no fusion, " making it difficult to understand what part of the cartilages contributed to carinal cartilage formation [15]. The most common type in the present study, the contribution of right bronchial cartilages, was recorded as 5% in the review [15]. In contrast, we found that the carinal cartilage was mainly composed of bronchial cartilage rather than the most inferior tracheal cartilage. About 79.3% of the dissected carinal cartilages in this study were composed of bronchial cartilages solely or with other components, and only 31.0% were made from tracheal cartilages (Fig. 2, Table 1).

Although carinal resection and reconstruction is still the standard surgical repair technique used to treat various tracheal diseases, including benign airway stenosis, lung or tracheal tumors, local patchy defects, and tracheal rupture, this can have complications such as anastomotic tension and poor cartilage healing. Therefore, a thorough anatomical understanding of the distal trachea, including the carina, is essential to address these challenges. Because the extent of the resection determines the anastomotic tension, knowledge of the contributions to the carinal cartilage can help surgeons decide the extent of cartilage resection.

During the development, a laryngotracheal diverticulum forms the trachea and two primary bronchial buds for the lungs and bronchi. The epithelium and glands of the trachea are differentiated from the endodermal lining of the laryngotracheal tube. The cartilage, connective tissue, and smooth muscle of the trachea originate from the splanchnic mesenchyme of the laryngotracheal tube. The primary bronchial buds differentiate into bronchi from the surrounding splanchnic mesenchyme [4, 12]. The connection of the bronchial buds and trachea enlarges to form the primordia of the main bronchi early in the fifth week of development, which is assumed to be related to development of the carina, although the specific details of carinal development have yet to be studied [12].

This study observed that the carinal cartilages were deviated from the midline in 4.37%, which might increase the risk of aspiration into the right bronchus [14].

Histologically, we found that the carina showed typical morphology/characteristics of the respiratory tract. However, extensive bundles of collagen fibers were observed under the respiratory epithelium layer. Kamel and colleagues reported the structures as extensive bundles of elastic fibers with Van-Gieson stain [9]. According to Kamel et al., the elastic fiber bundles continue to the bronchi and may be involved in elongation of the trachea and main bronchi during deep inspiration [5, 6].

## Conclusions

Although the carina has been regarded as a part of the distal trachea, the present study found that most of the carinal cartilages were composed of the most inferior tracheal ring or bronchial cartilage(s). The right bronchial cartilage was the most common contributor, and the left was the second most common contributor. The carinal is deviated slightly to the left of the midline in most specimens. On micro-CT, the carina’s cartilaginous makeup could be determined. Lastly, the term carina can be confused between authors. Therefore, we suggest that the terms carina and carinal cartilage be used.

## Data Availability

No datasets were generated or analysed during the current study.
